# A Longitudinal Nine-Year Study of the Molecular Epidemiology of Carbapenemase-Producing *Enterobacterales* Isolated From a Regional Hospital in Taiwan: Predominance of Carbapenemase KPC-2 and OXA-48

**DOI:** 10.3389/fmicb.2022.703113

**Published:** 2022-03-11

**Authors:** Tran Thi Thuy Duong, Ya-Min Tsai, Li-Li Wen, Hui-Chuan Chiu, Pek Kee Chen, Tran Thi Dieu Thuy, Pei-Yun Kuo, Jazon Harl Hidrosollo, Shining Wang, Yen-Zhen Zhang, Wei-Hung Lin, Ming-Cheng Wang, Cheng-Yen Kao

**Affiliations:** ^1^Institute of Microbiology and Immunology, School of Life Sciences, National Yang Ming Chiao Tung University, Taipei, Taiwan; ^2^Department of Clinical Laboratory, En Chu Kong Hospital, New Taipei City, Taiwan; ^3^Institute of Clinical Medicine, College of Medicine, National Cheng Kung University, Tainan, Taiwan; ^4^Division of Nephrology, Department of Internal Medicine, National Cheng Kung University Hospital, College of Medicine, National Cheng Kung University, Tainan, Taiwan; ^5^Institute of Clinical Pharmacy and Pharmaceutical Sciences, College of Medicine, National Cheng Kung University, Tainan, Taiwan

**Keywords:** carbapenem-resistant *Enterobacterales* (CRE), KPC-2, OXA-48, NDM, pulsed-field gel electrophoresis (PFGE), carbapenemase

## Abstract

Enterobacterales clinical isolates are now being resistant to clinically achievable concentrations of most commonly used antibiotics that makes treatment of hospitalized patients very challenging. We hereby determine the molecular characteristics of carbapenemase genes in carbapenem-resistant *Enterobacterales* (CRE) isolates in Taiwan. A total of 455 CRE isolates were identified between August 2011 to July 2020. Minimum inhibitory concentrations for selected carbapenems were tested using Vitek 2, and carbapenemase genes were determined using polymerase chain reaction in combination with sequencing. Phenotypic detection of carbapenemase was determined by modified carbapenem inactivation method (mCIM) and EDTA-modified carbapenem inactivation method (eCIM) to validate our PCR screening results. Pulsed-field gel electrophoresis (PFGE) was used to determine the clonality of carbapenemase-producing *Enterobacterales* (CPE) isolates, and the transferability of carbapenemase-carrying plasmids was determined by conjugation assays. A slight increase in carbapenem-resistant *E. coli* (CREC) was observed, however, the prevalence of carbapenem-resistant *K. pneumoniae* (CRKP) was steady, during 2011–2020. The dominant species among our CRE was *K. pneumoniae* (270/455, 59.3%), followed by *E. coli* (81/455, 17.8%), *Morganella morganii* (32/455, 7.0%), and *Enterobacter cloacae* (25/455, 5.5%). From 2011 to 2020, the total percentage of CPE increased steadily, accounting for 61.0% of CRE in 2020. Moreover, 122 of 455 CRE isolates (26.8%) were CPE. Among the CPE isolates, the dominant carbapenemase gene was *bla*_OXA–48–like_ (54/122, 44.3%), and the second most common carbapenemase gene was *bla*_KPC–2_ (47/122, 38.5%). The sensitivity and specificity for mCIM to detect carbapenemase in the 455 isolates were both 100% in this study. The PFGE results showed that 39 carbapenemase-producing *E. coli* and 69 carbapenemase-producing *K. pneumoniae* isolates carrying *bla*_KPC–2_ and/or *bla*_NDM–5_ could be classified into 5 and 12 clusters, respectively. In conclusion, our results showed an increase in CPE isolates in Taiwan. Moreover, the distribution of carbapenemase and antimicrobial susceptibility in CPE were associated with PFGE typing.

## Introduction

*Enterobacterales* are Gram-negative, facultatively anaerobic, non-spore-forming rods, and one of the most common causes of nosocomial infections. Successive studies have demonstrated increasing antibiotic resistance among clinical *Enterobacterales* isolates, and high proportions of *Enterobacterales* isolates are now non-susceptible to clinically achievable concentrations of most commonly used antibiotics, such as broad-spectrum cephalosporins (Iredell et al., 2016; De Oliveira et al., 2020). Carbapenems are considered effective antimicrobial options for the treatment of critically ill patients with a variety of bacterial infections due to their broadest spectrum among β-lactam antibiotics and their relative resistance to hydrolysis by most β-lactamases (Barry et al., 1985). However, carbapenem resistance rates in *Enterobacterales* isolates have increased worldwide over the past decade (Logan and Weinstein, 2017; Lutgring, 2019).

The presence of innate resistance mechanisms and the acquisition of clusters of foreign resistance genes (e.g., plasmid, transposon, or integron) that promote survival of *Enterobacterales* under antibiotic treatment and host selection pressures are associated with the rapid emergence of multidrug-resistant (MDR) or extensively drug-resistant (XDR) *Enterobacterales* worldwide (Iredell et al., 2016; Kopotsa et al., 2019). Reduced expression or mutations in porins, overexpression of efflux pumps, and the presence of β-lactamases, play a critical role in resistance to carbapenems (Poirel et al., 2004; Iredell et al., 2016; Sugawara et al., 2016; De Oliveira et al., 2020). The first reported plasmid-mediated carbapenemase gene, *K. pneumoniae* carbapenemase (*bla*_KPC_), was identified in *K. pneumoniae* in 2001 (Yigit et al., 2001) and became the predominant carbapenemase in *K. pneumoniae* (Chen L. et al., 2014; Chen et al., 2018). *bla*_NDM_ is the second most common carbapenemase found among carbapenem-resistant *Enterobacterales* (CRE) in China and is more prevalent in *E. coli* (Zhang et al., 2018). Therefore, the study aimed to investigate the molecular epidemiology of CRE in a regional hospital in Taiwan during 2011–2020.

## Materials and Methods

### Identification of Carbapenem-Resistant *Enterobacterales* Isolates

*Enterobacterales* were isolated at En Chu Kong Hospital (ECKH), from 2011 August to 2020 July. En Chu Kong hospital, located at Sanxia district, New Taipei city, is an approximate 500-bed capacity regional teaching hospital (include three buildings: Fuxing building, Zhongshan building, and outpatient department building). The ECKH provides comprehensive medical services from fetus to the elderly, from acute trauma to hospice care, and from precision medicine to community health. These isolates were identified in the clinical laboratory by colony morphology, Gram stain, biochemical tests, and the Vitek 2 system (bioMérieux, Marcy-l’Étoile, France) according to the manufacturer’s recommendations. Non-duplicate 27,585 *E. coli* and 11,582 *K. pneumoniae* were collected in this study. The susceptibility of *Enterobacterales* isolates to third-generation cephalosporins (ceftazidime or ceftriaxone, 30 μg/disc, BD BBL™ Sensi-Disc™, Sparks, MD, United States) was determined by the disk diffusion method on Mueller-Hinton (MH) agar plates according to the Clinical and Laboratory Standards Institute (CLSI) guidelines (M100-S30) (CLSI, 2020). Third-generation cephalosporin-resistant isolates were also tested for susceptibility to carbapenems, including imipenem, ertapenem, meropenem, and doripenem (10 μg/disc, BD BBL, United States). A total of 455 CRE isolates were identified and stored at −80°C in tryptic soy broth (TSB) containing 20% glycerol (v/v) until use.

### Carbapenemase Gene Detection

Bacterial genomic DNA was isolated from bacteria grown overnight at 37°C in 3°ml LB broth. The bacterial culture was centrifuged at 12,000 rpm for 1 min, and the supernatant was removed. Crude DNA extracts were obtained by suspending the pellet in 300 μl distilled water and boiling at 95°C for 10min, followed by centrifugation at 12,000 rpm for 5 min. The DNA-containing supernatant was transferred to a new eppendorf tube, and DNA samples were stored at 4°C until testing. PCR amplification for detection of β-lactamase genes (*bla*_KPC,_ *bla*_NDM,_ *bla*_IMP,_ *bla*_VIM,_ *bla*_OXA–48_, *bla*_GES_, *bla*_IMI_, *bla*_SME_, *bla*_SPM_, *bla*_SIM_, *bla*_DIM_, and *bla*_GIM_) was performed on a GeneExplorer Thermal Cycler (BIOER, China) with the Fast-Run™ 2× Taq Master Mix (Protech, Taipei, Taiwan). Primers and PCR procedures used for the detection of β-lactamase genes have been described in previous studies (Yigit et al., 2001; Ellington et al., 2007; Doyle et al., 2012; Mlynarcik et al., 2016). PCR products were analyzed by electrophoresis using 1.2% agarose gels in 0.5× Tris-borate-EDTA (TBE) buffer. Gels were stained with ethidium bromide (EtBr), and PCR products were visualized using UV transilluminator. Clinical *K. pneumoniae* isolates carrying *bla*_KPC_, *bla*_NDM_, *bla*_IMP_, *bla*_VIM_, and *bla*_OXA–48_ were used as PCR positive controls. The PCR products of *bla*_GES_, *bla*_IMI_, *bla*_SME_, *bla*_SPM_, *bla*_SIM_, *bla*_DIM_, or *bla*_GIM_, with relevant expected sizes were verified by sequencing due to the lack of relative control strains.

### Phenotypic Detection of Carbapenemase-Producing *Enterobacterales*

The modified carbapenem inactivation method (mCIM) and EDTA-modified carbapenem inactivation method (eCIM) were performed on CRE isolates according to the previous study to detect the presence of carbapenemase (Sfeir et al., 2019; Tsai et al., 2020). Briefly, a 1-μl loopful of bacteria was resuspended in a 2-ml tube containing TSB. Another 1-μl loopful of bacteria was resuspended in a 2-ml tube containing TSB supplemented with EDTA at a final concentration of 5 mM. A meropenem disk was placed in each tube, and the tubes were incubated at 35°C for 4 h ± 15 min. The disks were then removed and placed onto MH agar plates freshly plated with a 0.5 McFarland suspension of a carbapenem-susceptible *E. coli* strain ATCC 25922. Plates were incubated at 35°C for 16 to 20 h, and mCIM and eCIM results were interpreted as previously described (Sfeir et al., 2019). In accordance with CLSI guidelines (CLSI, 2020), *K. pneumoniae* ATCC BAA-1706 (carbapenemase negative), *K. pneumoniae* ATCC BAA-1705 (*bla*_KPC_ positive), and *K. pneumoniae* ATCC BAA-2146 (*bla*_NDM_ positive) were used as internal controls for mCIM and eCIM testing. The mCIM and eCIM tests were replicated by two independent investigators to ensure reproducibility.

### Determination of Minimum Inhibitory Concentrations

Minimum inhibitory concentrations (MICs) for cefmetazole, cefotaxime, ceftazidime, cefepime, imipenem, ertapenem, meropenem, amikacin, gentamicin, ciprofloxacin, levofloxacin, tigecycline, colistin, and trimethoprim for CPE isolates were determined with Vitek 2 using the card AST-N322 according to the manufacturer’s instructions. *E. coli* ATCC 25922 was used as a quality control strain. Antibiotic susceptibility (except tigecycline) was interpreted according to CLSI guidelines (M100-S30) (Zhang et al., 2018). The results of tigecycline susceptibility were interpreted according to the breakpoints of the U.S. Food and Drug Administration (FDA) (≥ 8.0 μg/ml, resistant; 4.0 μg/ml, intermediate; ≤ 2.0 μg/ml, susceptible).

### Pulsed-Field Gel Electrophoresis (PFGE)

Pulsed-field gel electrophoresis was performed to determine the clonality of CPE isolates according to a previous study (Li et al., 2018). Briefly, PFGE of *Xba*I-digested genomic DNA was performed using a CHEF Mapper XA instrument (Bio-Rad Laboratories, Inc., Hercules, CA, United States) with the following parameters: separation on a 1% agarose gel (Seakem Gold agarose; FMC Bio Products) in 0.5× TBE buffer for 19 h at 14°C with pulse times ranging from 5 to 35 s at 6 V/cm. Gels were stained with EtBr and photographed with UV transillumination. PFGE profiles were analyzed and compared using the GelCompar II software, version 2.0 (Unimed Healthcare, Inc., Houston, TX, United States). The PFGE patterns were interpreted according to a previous study (Bando et al., 2009) and the isolates having > 80% pattern similarity were assigned to the same cluster.

### Conjugation Experiments

The liquid mating-out assay was performed to transfer carbapenemase genes from CPE isolates to the rifampicin- and streptomycin-resistant *E. coli* C600 strain as previously described (Kao et al., 2016). All isolates tested were sensitive to rifampicin or streptomycin at the concentration of 256 μg/ml. Therefore, transconjugants were selected on LB plates with 256 μg/ml rifampicin (Sigma-Aldrich, United States) or 256 μg/ml streptomycin (Sigma-Aldrich, United States) in combination with 1 μg/ml meropenem. The conjugation assay was performed in triplicate to determine the transferability of the plasmid.

### Statistical Analysis

A Cochran–Armitage test was used to evaluate trends in CREC, CRKP, CPE, CPEC, and CPKP over time. Statistical analyses were performed using the JMP software (SAS Institute Inc., Cary, NC, United States). A *p*-value < 0.05 is statistically significant.

## Results

### Identification of Carbapenem-Resistant *Enterobacterales*

**FIGURE 1:**
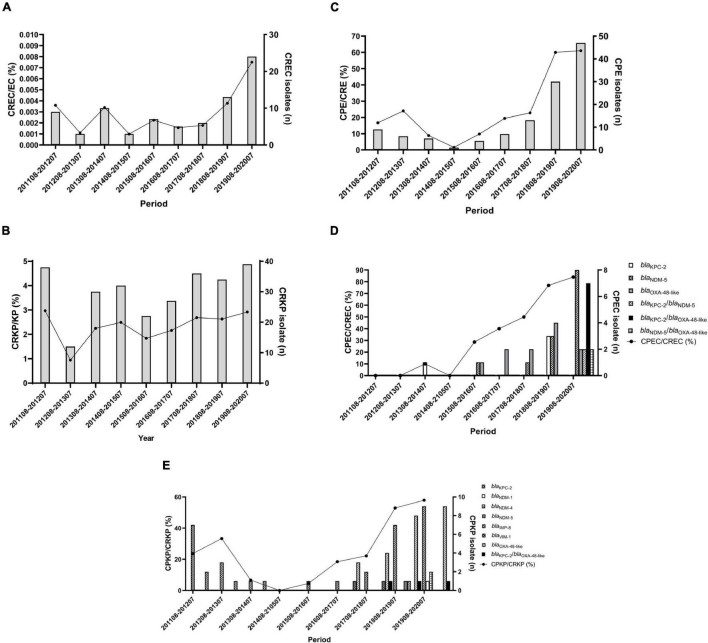
Distribution of carbapenemase-producing *Enterobacterales* during 2011–2020. **(A)** Annual proportions and numbers of carbapenem-resistant *E. coli* among all *E. coli*. **(B)** Annual proportions and numbers of carbapenem-resistant *K. pneumoniae* among all *K. pneumoniae*. **(C)** Annual proportions and numbers of carbapenemase-producers among CRE. **(D,E)** Annual proportions and numbers of carbapenemase-producers among carbapenem-resistant *E. coli* **(D)** and *K. pneumoniae* **(E)**. The percentage of isolates is plotted as a line graph on the primary axis while the number of isolates is plotted as bars on the secondary axis.

The dominant species among our CRE was *K. pneumoniae* (270/455, 59.4%), followed by *E. coli* (80/455, 17.6%), *Morganella morganii* (32/455, 7.0%), and *Enterobacter cloacae* (25/455, 5.5%) (Table 1). Thus, 0.003% (80/27,585) *E. coli* and 2.323% (270/11,582) *K. pneumoniae* showed resistance to carbapenem. A slight increase in carbapenem-resistant *E. coli* (CREC) was observed during 2011–2020 (*p* > 0.05) (Figure 1A). In contrast, the prevalence of carbapenem-resistant *K. pneumoniae* (CRKP) was steady (*p* > 0.05) (Figure 1B). In addition, 205 (44.9%) and 169 (37.0%) CRE strains were isolated from urine and sputum, respectively (Table 1). CRKP was most frequently isolated from sputum (127/270, 47.0%), followed by urine (107/270, 39.6%). In contrast, CREC was most frequently isolated from urine (54/80, 67.5%), followed by sputum (12/80, 15.0%) (Table 1).

**TABLE 1:** Source of clinical specimens and bacterial species of 455 non-duplicate CRE.

	Clinical specimens’ source											No. of isolates
	Urine	Sputum	Wound pus	Bronchial washing	Abscess	Blood	Vaginal discharge	Ear discharge	Catheter tip	Body fluid	Nasal	
*Citrobacter freundii*	1	0	0	0	0	1	0	0	0	0	0	2
*Citrobacter koseri*	3	2	1	0	0	1	0	0	0	0	0	7
*Citrobacter youngae*	1	0	0	0	0	0	0	0	0	0	0	1
*Enterobacter aerogenes*	1	6	1	0	0	0	0	0	0	0	1	9
*Enterobacter cloacae*	10	9	0	1	1	3	0	0	1	0	0	25
*Escherichia coli*	54	12	3	3	2	5	1	0	0	0	0	80
*Escherichia hermannii*	0	1	0	0	0	0	0	0	0	0	0	1
*Klebsiella oxytoca*	1	1	0	0	0	0	0	0	0	0	0	2
*Klebsiella pneumoniae*	107	127	8	15	0	11	0	0	0	2	0	270
*Morganella morganii*	13	4	9	2	0	3	0	1	0	0	0	32
*Providencia rettgeri*	6	0	0	0	0	2	0	0	0	0	0	8
*Providencia stuartii*	6	5	1	0	0	0	0	0	0	0	0	12
*Serratia marcescens*	2	2	1	0	0	1	0	0	0	0	0	6
no. of isolates	205	169	24	21	3	27	1	1	1	2	1	455

### Distribution of Carbapenemase Genes in Carbapenem-Resistant *Enterobacterales*

PCR was used to detect the presence of carbapenemase genes, and the results showed that 122 of 455 CRE isolates (26.8%) were CPE (Table 2). No carbapenemase genes were detected in carbapenem-resistant *Providencia rettgeri*, *Providencia stuartii*, and *Serratia marcescens*. In addition, no GES-, IMI-, SME-, SPM-, SIM-, DIM-, and GIM-producers were identified in our CRE isolates. Overall, the dominant carbapenemase gene was *bla*_OXA–48–like_ (54/122, 44.3%) among CPE isolates, and the second most common carbapenemase gene was *bla*_KPC–2_ (47/122, 38.5%) (Table 2). In addition, we found metallo-carbapenemases NDM, IMP, and VIM in 23 (18 *bla*_NDM–5_, 3 *bla*_NDM–1_, and 2 *bla*_NDM–4_), 9 (9 *bla*_IMP–8_), and 4 (*bla*_VIM–1_) CPE isolates, respectively (Table 2). Importantly, 15 CPE isolates carrying more than one carbapenemase gene were identified during 2017–2020. Seven *E. coli* isolates and 3 *K. pneumoniae* isolates had both *bla*_OXA–48–like_ and *bla*_KPC–2_ (Table 2). Coexistence of *bla*_KPC–2_/*bla*_NDM–5_ and *bla*_OXA–48–like_/*bla*_NDM–5_ was found in 2 and 2 carbapenem-resistant *E. coli*, respectively. One *Klebsiella oxytoca* isolate had both *bla*_VIM–1_ and *bla*_NDM–1_ (Table 2).

**TABLE 2:** The distribution of carbapenemase genes among 455 non-duplicate CRE.

	Carbapenemase genes											No. of isolates
	*bla*_KPC–2_	*bla*_NDM–1_	*bla*_NDM–4_	*bla*_NDM–5_	*bla*_IMP–8_	*bla*_VIM–1_	*bla*_OXA–48–like_	*bla*_KPC–2_ *bla*_NDM–5_	*bla*_KPC–2_ *bla* _OXA–48–like_	*bla*_NDM–5_ *bla* _OXA–48–like_	*bla*_NDM–1_ *bla*_VIM–1_	
*Citrobacter freundii*	0	0	0	0	1	0	0	0	0	0	0	1
*Citrobacter koseri*	0	0	0	0	0	0	4	0	0	0	0	4
*Citrobacter youngae*	0	0	0	0	0	0	1	0	0	0	0	1
*Enterobacter aerogenes*	0	1	0	0	0	1	0	0	0	0	0	2
*Enterobacter cloacae*	0	0	0	0	2	0	1	0	0	0	0	3
*Escherichia coli*	4	0	0	13	0	0	11	2	7	2	0	39
*Escherichia hermannii*	0	0	0	0	1	0	0	0	0	0	0	1
*Klebsiella oxytoca*	0	0	0	0	0	0	0	0	0	0	1	1
*Klebsiella pneumoniae*	31	1	2	1	5	2	24	0	3	0	0	69
*Morganella morganii*	0	0	0	0	0	0	1	0	0	0	0	1
no. of isolates	35	2	2	14	9	3	42	2	10	2	1	122

Among the CRE isolates, 39 (39/80, 48.8%) *E. coli* and 69 (69/270, 25.6%) *K. pneumoniae* isolates were CPE. The dominant carbapenemase gene was *bla*_OXA–48–like_ (20/39, 51.3%) among carbapenemase-producing *E. coli* (CPEC) isolates, and the second most common carbapenemase gene was *bla*_NDM–5_ (17/39, 43.6%). In contrast, *bla*_KPC–2_ was found dominant among carbapenemase-producing *K. pneumoniae* (CPKP) isolates (34/69, 49.3%), followed by *bla*_OXA–48–like_ (27/69, 39.1%) (Table 2).

### Modified Carbapenem Inactivation Method/EDTA-Modified Carbapenem Inactivation Method Phenotypic Detection of Carbapenemase Producer

It was previously reported that eCIM in combination with the mCIM is efficient for identifying CPE (Sfeir et al., 2019). Therefore, phenotypic detection mCIM/eCIM was performed on our 455 CRE isolates to validate PCR results for carbapenemase gene detection. In this study, the sensitivity and specificity for the mCIM to detect carbapenemase in 455 CRE isolates were both 100% which is consistent with our previous report (Tsai et al., 2020).

Interestingly, we found that four isolates containing metallo-carbapenemase NDM-5 and non-metallo-carbapenemases (OXA-48 or KPC-2) showed inconsistent mCIM/eCIM results (Table 3). *E. coli* isolate 488, an NDM-5 and OXA-48 producer, showed a false-negative result in mCIM/eCIM (Table 3). In contrast, *E. coli* isolate 514 with *bla*_NDM–5_ and *bla*_OXA–48_ showed a positive mCIM/eCIM result (Table 3). In addition, *E. coli* 571 and 572 with *bla*_KPC–2_/*bla*_NDM–5_ also showed positive results by mCIM/eCIM (Table 3).

**TABLE 3:** Characteristics of three isolates that contained both metallo-carbapenemases and non-metallo-carbapenemases.

		MIC (μ g/ml)			Disc zone (mm)						Phenotypic detection	
Isolate	Carbapenemase	IPM	ETP	MEM	IPM	ETP	MEM	DOP	mCIM	eCIM	mCIM	eCIM
*E. coli* 488^a^	*bla*_OXA–48_/*bla*_NDM–5_	≥16	≥8	8	6	6	6	6	6	6	+	–
*E. coli* 514	*bla*_OXA–48_/*bla*_NDM–5_	≥16	≥8	≥16	16	12	15	16	6	23	+	+
*E. coli* 571	*bla*_KPC–2_/*bla*_NDM–5_	≥16	≥8	≥16	13	9	12	14	6	20	+	+
*E. coli* 572	*bla*_KPC–2_/*bla*_NDM–5_	8	≥8	8	12	8	12	11	6	20	+	+

*^a^The characteristics of isolate E. coli 488 were reported in our previous study (Tsai et al., 2020). IPM, imipenem; ETP, ertapenem; MEM, meropenem; DOP, doripenem.*

### Increase in Carbapenemase-Producing *E. coli* and *K. pneumoniae* During 2011–2020

From 2011 to 2020, the total percentage of CPE increased steadily, accounting for 61.0% of CRE in 2020 (16.7% in August 2011–July 2012) (*p* < 0.0001) (Figure 1C). *E. coli* and *K. pneumonia*e isolates were dominant in our CPE collection (Table 2), so we aimed to further characterize the molecular epidemiology of CPEC and CPKP isolates. Among CPEC isolates, we found a dramatic increase in *bla*_NDM–5_ and *bla*_KPC–2_/*bla*_OXA–48–like_ in 2020 (*p* < 0.0001) (Figure 1D). In contrast to CPEC, *bla*_KPC–2_ and *bla*_OXA–48–like_ were predominant in CPKP isolates in 2020 (*p* < 0.0001) (Figure 1E).

### Pulsed-Field Gel Electrophoresis Typing of Carbapenemase-Producing *E. coli* and *K. pneumoniae*

**FIGURE 2:**
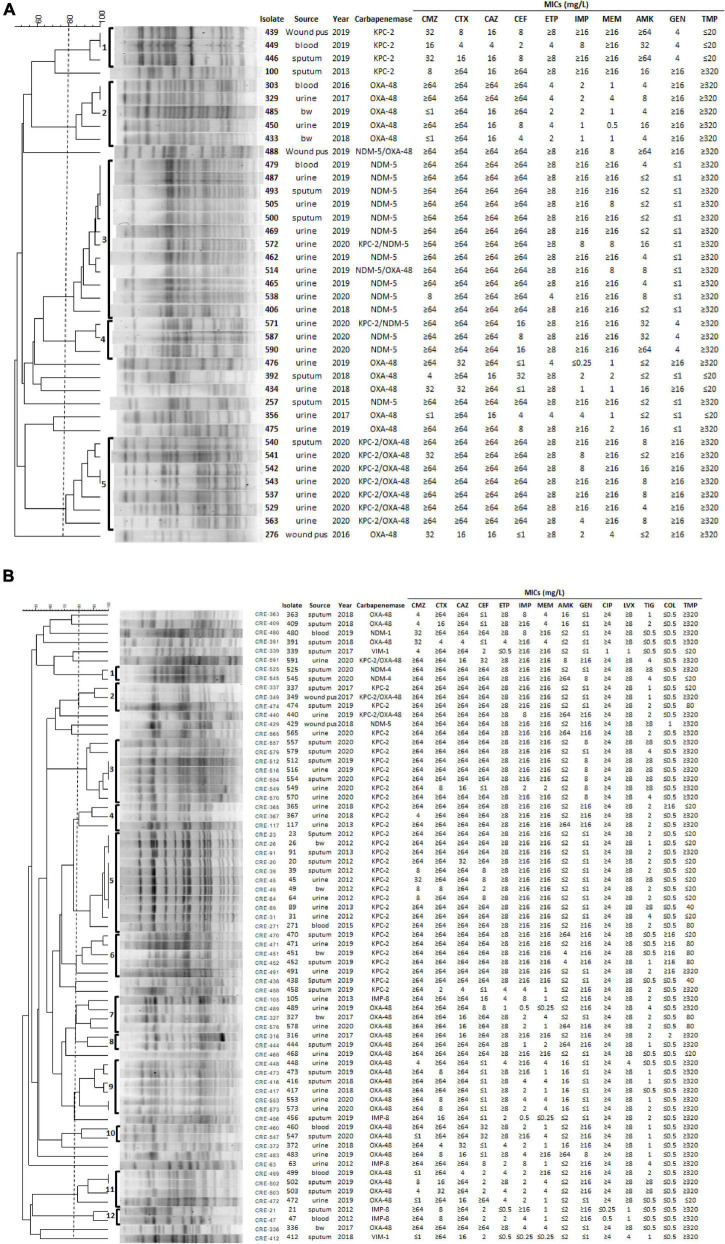
PFGE, the origin of isolate, year, MICs of antibiotics, and carbapenemase genes in 39 *E. coli* **(A)** and 69 *K. pneumoniae* **(B)** isolates. **(A)** All CPEC had the following MICs of antibiotics: ampicillin ≥ 32 μg/ml, piperacillin > 128 μg/ml, cefazolin ≥ 64 μg/ml, ciprofloxacin ≥ 4 μg/ml, levofloxacin ≥ 8 μg/ml, tigecycline ≤ 0.5 μg/ml, and colistin ≤ 0.5 μg/ml. **(B)** All CPKP had MICs of the antibiotics as follows, ampicillin ≥ 32 μg/ml and cefazolin ≥ 64 μg/ml. bw, bronchial washing; CMZ, cefmetazole; CTX, cefotaxime; CAZ, ceftazidime; CEF, cefepime; IPM, imipenem; ETP, ertapenem; MEM, meropenem; AMK, amikacin; GEN, gentamicin; CIP, ciprofloxacin; LVX, levofloxacin; TIG, tigecycline; COL, colistin; TMP, trimethoprim.

The clonality of 39 CPEC and 69 CPKP isolates carrying carbapenemase KPC-2, NDM, and OXA-48 was further determined by PFGE (Figure 2). The PFGE patterns of 30 *E. coli* CPE isolates were assigned to five clusters based on > 80% pattern similarity (Figure 2A). All isolates in cluster 1 (*n* = 3) contained *bla*_KPC–2_, isolates in cluster 2 (*n* = 5) contained *bla*_oxa–48_, and isolates in cluster 5 (*n* = 7) contained both *bla*_KPC–2_ and *bla*_oxa–48_ (Figure 2A). In contrast, isolates from clusters 3 (*n* = 12) and 4 (*n* = 3) contained *bla*_NDM–5_ (two isolates contained *bla*_NDM–5_ and *bla*_KPC–2_; one isolate contained *bla*_NDM–5_ and *bla*_oxa–48_) (Figure 2A). Interestingly, isolates belonging to clusters 2 or 5 were resistant to gentamycin (Figure 2A). Although all 39 CPEC were resistant to ciprofloxacin (MIC ≥ 4 μg/ml) and levofloxacin (MIC ≥ 8 μg/ml), these isolates were sensitive to tigecycline (MIC ≤ 0.5 μg/ml) and colistin (MIC ≤ 0.5 μg/ml) (Figure 2A).

The PFGE patterns of 69 CPKP isolates were assigned to 12 clusters based on > 80% pattern similarity (Figure 2B). All isolates in clusters 2 (*n* = 3), 3 (*n* = 7), 4 (*n* = 3), 5 (*n* = 11), and 6 (*n* = 5) contained *bla*_KPC–2_, whereas isolates in clusters 8 (*n* = 2), 9 (*n* = 6), 10 (*n* = 2), and 11 (*n* = 4) contained *bla*_oxa–48_ (Figure 2B). Clusters 1 (*n* = 2) and 12 (*n* = 2) isolates contained *bla*_NDM–4_ and *bla*_IMP–8_, respectively (Figure 2B). Only isolates in cluster 12 were susceptible to both ciprofloxacin and levofloxacin (Figure 2B). In addition, 6 and 11 CPKP were resistant to colistin (MIC ≥ 4 μl/ml) and tigecycline (MIC ≥ 8 μl/ml), respectively (Figure 2B). The PFGE results indicate that the distribution of carbapenemase and antimicrobial susceptibility in CPE were associated with PFGE typing (Figure 2).

### Carbapenemase Transfer and Plasmid Analysis

A total of 37 CPEC and 53 CPKP isolates carrying carbapenemase were further analyzed with conjugation assays to determine whether there were horizontally spread carbapenemase-carrying plasmids in Taiwan (18 CPE isolates showed resistance to rifampicin were excluded in this assay). Transfer of carbapenemase gene by conjugation to *E. coli* C600 was successful in 4 NDM-5-producing CPEC (isolates 257, 462, 500, and 505) and 1 IMP-8-producing CPKP (isolate 21). However, all *bla*_KPC–2_- and *bla*_OXA–48_-carrying plasmids did not show transferability.

## Discussion

In this study, we isolated 455 CRE isolates from a regional teaching hospital in Taiwan (2011 August to 2020 July) and present their characteristics. Our results showed that 122 of 455 CRE isolates were CPE (Table 2). No carbapenemase genes were detected in our carbapenem-resistant *P. rettgeri*, *P. stuartii*, and *S. marcescens*. However, we could not rule out the presence of other carbapenemases in these isolates.

The dominant carbapenemase gene among our CPE isolates was *bla*_OXA–48_, and the second most common carbapenemase gene was *bla*_KPC–2_ (Table 2). Chiu et al. (2018) showed a sharp increase in the annual prevalence rate of OXA-48-like producers among Taiwanese CPE isolates between 2012 and 2015. In 2017, *bla*_OXA–48_ was detected in 18.2% of CPE in Taiwan (Jean et al., 2018). Moreover, Wu et al. (2021) reported that the *bla*_KPC–2_ was the most common carbapenemase gene in CRKP isolated from patients with bacteremia at a hospital in northern Taiwan from 2013 to 2018. A 22-year (1998–2019) observation to determine the evolution of carbapenemase genes in *K. pneumoniae* in Taiwan discovered that the endemicity has changed from *bla*_IMP–8_, *bla*_NDM–1_, and *bla*_VIM–1_ to the most common *bla*_KPC–2_ and rapidly emerging *bla*_OXA–48_ (Lai and Yu, 2021). These results are consistent with our finding that the distribution of *bla*_OXA–48–like_ was dramatically increased after 2018. Therefore, whether there is a circulation of OXA-48-producing plasmids/isolates in Taiwan is worth continually monitoring. Surprisingly, a very low conjugation rate of carbapenemase genes-carrying plasmids was observed in this study. Therefore, taxonomic relatedness and recipient strain used for conjugation tests may limit the conjugation in liquid matings (Chen Y. T. et al., 2014; Alderliesten et al., 2020).

Interestingly, the data from Surveillance of Multicentre Antimicrobial Resistance in Taiwan (SMART) with a multicenter collection of bacteremic isolates of *E. coli* (*n* = 423) and *K. pneumoniae* (*n* = 372) showed the carbapenem resistance rates were 1.2% (5/423) in *E. coli* and 7.5% (28/372) in *K. pneumoniae* (Liu et al., 2020). Moreover, carbapenemase genes were detected in 67.8% *K. pneumoniae* isolates (19/28). Among the CRKP isolates, 57.1% (16/28) harbored *bla*_KPC_ (Liu et al., 2020). However, in 2019, we found OXA-48-like was the dominant carbapenemase in CRKP. These results suggested the difference in the distribution of carbapenemase genes in CRKP isolated from different specimens and regions. Moreover, previous studies showed that most KPC-2 producers in CRKP were ST11 (Liu et al., 2020; Wu et al., 2021). Therefore, the ST type of OXA-48-like producing CRKP is worth further investigating.

We also found a dramatic increase in *bla*_NDM–5_ and *bla*_KPC–2/blaOXA–48–like_ from 2018 to 2020 in CREC when compared to the years before 2018 (Figure 1). Huang et al. (2021) reported an increase of NDM-producing *E. coli* in northern Taiwan during 2016 to 2018. Importantly, in all five *bla*_NDM–5_-positive isolates, the *bla*_NDM–5_ gene was located in a ∼46 kb IncX3 plasmid that were nearly identical to each other (Huang et al., 2021). These five *bla*_NDM–5_-containing plasmids are similar to pP785-NDM5 from China (Ho et al., 2018). These results suggest the dissemination of a specific IncX3 *bla*_NDM–5_-containing plasmid in Taiwan. Seven CPEC isolates having *bla*_KPC–2/blaOXA–48–like_ were collected from August 2019 to July 2020 in this study. The clonality of these seven strains remain unclear and worth investigating to determine whether is a specific CPEC clone outbreak in the hospital.

In addition, we found 15 CPE isolates carrying more than one carbapenemase gene during 2017–2020 (Table 2). Whether these carbapenemase genes were located on a single plasmid is worth investigating. We also found that four isolates containing metallo-carbapenemase NDM-5 and non-metallo-carbapenemases (OXA-48 or KPC-2) showed inconsistent mCIM/eCIM results (Table 3). These results raised the possibility that different expression levels of carbapenemase genes were present in these isolates, thus affecting the phenotypic detection of mCIM/eCIM. In addition, it remains to be investigated whether the genotypes of the carbapenemase genes in these isolates affect their enzymatic activity.

The emergence of CRE strains that also exhibit resistance to colistin and tigecycline has become a major clinical concern, as these two antibiotics are used as first-line for the treatment of CRE infections (Doi, 2019). Our results showed that all CPEC were sensitive to colistin and tigecycline (Figure 2A), while 6 and 11 CPKP were resistant to colistin (MIC ≥ 4 μl/ml) and tigecycline (MIC ≥ 8 μl/ml), respectively (Figure 2B). The mechanisms responsible for colistin and tigecycline resistance of these CPKP isolates remain to be studied. In addition, all CPKP isolates (*n* = 5) in cluster 6 were resistant to colistin and 5 of 7 isolates in cluster 3 were resistant to tigecycline (Figure 2B). The characteristics of the isolates in clusters 3 and 6 are worthy of future study.

Hentschke et al. (2010) demonstrated the induction of AcrAB-mediated multidrug resistance by prior treatment with multiple antibiotics. Kanwar et al. (2018) also revealed the occurrence of treatment-emergent colistin-resistant KPC-producing *K. pneumoniae* after 8 days of colistin-based combination therapy due to disruption of *mgrB*. Studies with clinical isolates provided evidence of an association between the emergence of colistin and tigecycline resistance in CRKP and frequent antibiotic use (van Duin et al., 2014; Du et al., 2018; Kanwar et al., 2018). Therefore, it is worth investigating whether the patient received long-term antibiotics, including tigecycline or colistin, after the diagnosis of CRKP infection, inducing resistance in this study.

Salipante et al. (2015) reported that whole-genome sequencing (WGS) has significantly improved resolving power for strain typing compared to PFGE. However, the present barriers to the universal adoption of WGS by clinical laboratories include relatively high costs of instrumentation and a lack of bioinformatic expertise (Salipante et al., 2015). Therefore, in this study, we performed PFGE to determine the clonality of 39 CPEC and 69 CPKP isolates carrying carbapenemase KPC-2, NDM, and OXA-48.

In conclusion, our longitudinal collection of isolates showed the increase of CPE in CRE isolates in Taiwan during 2011–2020, and the dominant carbapenemase gene was *bla*_OXA–48–like_, followed by *bla*_KPC–2_, among our CPE isolates. Moreover, we found the carbapenemase distribution and antimicrobial susceptibility in CPE were associated with PFGE typing. Although the analysis of our study was restricted to a single hospital as opposed to population-based, the continued epidemiological surveillance and control of antimicrobial prescribing and consumption would reduce the prevalence of drug-resistant organisms and the spread of antibiotic resistance.

## Data Availability Statement

The original contributions presented in the study are included in the article/supplementary material, further inquiries can be directed to the corresponding author.

## Author Contributions

Y-MT, H-CC, and L-LW contributed to the collection of isolates. TD, PC, Y-MT, H-CC, P-YK, TT, JH, SW, Y-ZZ, W-HL, and M-CW performed the experiments and interpreted the results of bacterial identification, antibiotic susceptibility tests, mCIM/eCIM tests, carbapenemases detection, and conjugation assays. C-YK was responsible for data analysis, manuscript writing, designed the study, and responsible for overall management and planning. All authors read and approved the final manuscript.

## Conflict of Interest

The authors declare that the research was conducted in the absence of any commercial or financial relationships that could be construed as a potential conflict of interest.

## Publisher’s Note

All claims expressed in this article are solely those of the authors and do not necessarily represent those of their affiliated organizations, or those of the publisher, the editors and the reviewers. Any product that may be evaluated in this article, or claim that may be made by its manufacturer, is not guaranteed or endorsed by the publisher.
